# Remarks on Porcelain Work

**Published:** 1859-09

**Authors:** M. Loomis


### REMARKS ON PORCELAIN WORK,

BY M. LOOMIS.

*Delivered before the Am. Dental Convention, Aug. 4, 1859.*

MR. PRESIDENT:—Three years ago, I stated in this Convention that I had a plan for making sets of artificial teeth by substituting mineral for metals; and from the same kind of percelain that mineral teeth are made. I construct an artificial denture entire,—teeth and plate continuous and of the same material.

At that time I had some three years' experience in making and testing the method, and was firm in my belief of its pre-eminent superiority; and during the three years which have followed since then, many others as well as myself, have been constantly engaged in making and applying the same kind of teeth, and I must say, that in all instances patients have attested their utmost satisfaction, frequently returning to express their appreciation and gratitude, and in scarcely *an instance*, during the past five years, has a patient returned to me at all dissatisfied, or with the teeth in any way injured. And I do say, most emphatically, that where this plan is adopted the work of repairing will be abandoned *in toto*, for artificial teeth will no longer wear out, and the ratio of loss or destruction by accident is not more than one in fifty.

Notwithstanding, a great many discouraging things have been said about this method, but they originate either in prejudice, or ignorance of its real merits; for where a thing is made so beautiful and so absolutely perfect as these sets are, and are so entirely pure and clean in fulfilling their purpose, *and fulfilling that purpose so well*, and never wearing out or giving way, as theory and trial have abundantly demonstrated, it certainly leaves nothing to desire in an artificial denture. I have now watched and tested it, as I said, in practical operation for six years, and I do not know of a single objection to be brought against it, other than that it *is artificial.*

Persons for whom it has been made and who have tried different kinds, and who measure its usefulness and value by the test of rigid experience, are unanimous in declaring a very great preference for this; and those who have lost the teeth that nature gave them can best appreciate what it is to have an artificial perfection.

There may be,—yes, there *are* those whose pecuniary interests are adverse to the introduction of this style of work. This fact, together with prejudice and an ignorance of its merits, and bearing also the burden of Letters Patent, form a heavy clog to its progress, and though it is gaining ground slowly—as all really valuable things generally do, yet it *is* gaining nevertheless; and, mark my words, the "Mineral Plate" is gaining that ascendancy which it never will yield up, and though the time may seem remote to its enemies, and those who have little faith in it, yet it is as sure of universal adoption as the sun is to rise and set in coming years; for it is based upon common sense and natural principles, pointing out such a success with mathematical accuracy.

I am not here to decry other styles and other practices, but to say a very few words in a very short time in relation to the merits of a practice, as yet comparatively little understood or appreciated by our profession.

I will match my style for sets of teeth, in point of artistic beauty, durability and practical usefulness with any thing which our profession has yet known, *and will challenge the* WORLD to produce any thing which will compare favorably with it.

In simplicity of construction, in expensiveness and durability, it certainly is the great desideratum of artificial work. Will it be contended that it is difficult to make? We who are familiar with its construction, find it to be a more refined order of work, more artistic, less laborious, and by far the easiest method yet devised to make a reliable set of artificial teeth; and, if need be, the best article which art can thus produce can be afforded to the poor, at a price below all competition. But its construction, like all other nice manipulations requires judgment, skill and practice. And although many who have long been in practice may be adverse to serve an additional pupilage to acquire the fullness of their profession, yet I hope no one will carry the dislike so far as to be content with our present knowledge, or to deem a thing worthless simply because it has not transpired before. Let me say in conclusion, gentlemen, *try* this style for sets of teeth, put them into the mouth *and test them severely*, and you will believe what I say, that it is the *ne plus ultra* in this department of dental science, for I am taught by that experience which does not deceive, and if I am enthusiastic, it is because I have abundant reason to be, from its complete success.

